# Carbonic anhydrase IX is a marker of hypoxia and correlates with higher Gleason scores and ISUP grading in prostate cancer

**DOI:** 10.1186/s13000-016-0495-1

**Published:** 2016-05-25

**Authors:** Maria Raffaella Ambrosio, Claudia Di Serio, Giovanna Danza, Bruno Jim Rocca, Alessandro Ginori, Igor Prudovsky, Niccolò Marchionni, Maria Teresa del Vecchio, Francesca Tarantini

**Affiliations:** Department of Medical Biotechnology, Section of Pathology, University of Siena, Via delle Scotte 6, 53100 Siena, Italy; Department of Clinical and Experimental Medicine, Research Unit of Medicine of Ageing, University of Florence, Florence, Italy; Department of Clinical Physiopathology, Endocrine Unit, University of Florence, Florence, Italy; Section of Pathology, Ospedale di Circolo di Busto Arsizio, Presidio Ospedaliero di Saronno, Saronno, Italy; Center for Molecular Medicine, Maine Medical Center Research Institute (MMCRI), Scarborough, ME USA; Department of Medicine, Science and Neurosciences, University of Siena, Siena, Italy

**Keywords:** Prostate cancer, Carbonic anhydrase, HIF-1α, Hypoxia, Gleason score

## Abstract

**Background:**

Carbonic anhydrase IX is a member of α-carbonic anhydrases that is preferentially expressed in solid tumors. It enables bicarbonate transport across the plasma membrane, neutralizing intracellular pH and conferring to cancer cells a survival advantage in hypoxic/acidic microenvironments. Overexpression of carbonic anhydrase IX in cancer tissues is regulated by hypoxia inducible factor 1α − mediated transcription and the enzyme is considered a marker of tumor hypoxia and poor outcome. The role of carbonic anhydrase IX in prostate cancer has not been fully clarified and controversy has arisen on whether this enzyme is overexpressed in hypoxic prostate cancer tissues.

**Methods:**

We analyzed the expression of carbonic anhydrase IX and hypoxia inducible factor 1α in two prostate cancer cell lines, LNCaP and PC-3, and in 110 cancer biopsies, by western blotting and immunocyto/histochemistry.

**Results:**

In LNCaP and PC-3 cells, carbonic anhydrase IX was mostly cytoplasmic/nuclear, with very limited membrane localization. Nuclear staining became stronger under hypoxia. When we analyzed carbonic anhydrase IX expression in human prostate cancer biopsies, we found that protein staining positively correlated with hypoxia inducible factor 1α and with Gleason pattern and score, as well as with the novel grading system proposed by the International Society of Urological Pathology for prostate cancer. Once more, carbonic anhydrase IX was mainly cytoplasmic in low grade carcinomas, whereas in high grade tumors was strongly expressed in the nucleus of the neoplastic cell. An association between carbonic anhydrase IX expression level and the main clinic-pathological features involved in prostate cancer aggressiveness was identified.

**Conclusions:**

There was a statistically significant association between carbonic anhydrase IX and hypoxia inducible factor 1α in prostate cancer tissues, that identifies the enzyme as a reliable marker of tumor hypoxia. In addition, carbonic anhydrase IX expression positively correlated with prostate cancer grading and staging, and with outcome, suggesting that the protein may be an independent prognosticator for the disease. The nuclear translocation of the enzyme in hypoxic cancer cells may epitomize a biological switch of the tumor towards a less favorable phenotype.

## Background

Carbonic anhydrase (CA) IX is a membrane-associated glycoprotein, belonging to the family of α-carbonic anhydrases that catalyze the reversible hydration of carbon dioxide to bicarbonate ions and protons [1]. Sixteen isoforms of CA have been identified in humans: five are cytosolic (CA I, II, III, VII and XIII), five are membrane-bound (CA IV, IX, XII, XIV and XV), two reside in the mitochondria (CA VA and VB) and one is secreted in milk and saliva (CA VI). Moreover, three non-catalytic isoforms have been described and designated CA-related proteins (CARP VIII, X and XI) [2]. The catalytic-competent isoforms perform many biological functions, involving pH regulation and ion transport in many organs [3]. Of all isoforms, CA IX is preferentially expressed in solid tumors and its presence in normal tissues is limited to the gastrointestinal tract where the enzyme is implicated in cell proliferation and differentiation [4, 5]. CA IX consists of a short intracellular tail, a transmembrane region, a large extracellular domain (ectodomain) which retains the catalytic activity and displays a unique proteoglycan-like domain which seems to favor cell-adhesion processes. The protein also comprises a signal peptide [6].

The overexpression of CA IX in cancer tissues is strongly regulated by hypoxia, through the hypoxia-inducible factor (HIF)-1 mediated transcription [7, 8]. Indeed, the ability of the enzyme to neutralize intracellular pH, by facilitating bicarbonate transport across the plasma membrane, confers to cancer cells a survival advantage when they are exposed to hypoxic and acidic microenvironments [9]. Moreover, by intensifying the extracellular acidosis, CA IX facilitates the activation of proteases that degrade the extracellular matrix, stimulating migration and invasion of the surrounding tissues [10].

In addition to the hypoxia-induced modulation of gene transcription, the level of CA IX detected on cell surface is dependent on two additional mechanisms of regulation: endocytosis and ectodomain (ECD) shedding [3]. Endocytosis is also positively controlled by hypoxia [11] while ECD shedding seems to be supported by at least two distinct metalloproteases [12]. Whether ECD released into the extracellular space is inactive or is biologically active is still unknown.

Hypoxia is a common feature of prostate cancer (PC) and foreshadows a poor prognosis [13]. The first indication that carbonic anhydrases may be relevant for PC cell survival derives from the work of Supuran *et al*. that described the antitumor effect of CA inhibitors on several cancer cell lines, including prostate cancer [14, 15]. Recently, Fiaschi *et al*. clearly demonstrated the presence of CA IX in three different PC cell lines, PC-3, Du145 and LNCaP [10]. In human tissues, a study conducted in 9 men undergoing needle oxygen measurements and biopsy of tumor bearing prostate glands, demonstrated CA IX positivity in areas of hypoxia [16]. On the contrary, in an immunohistochemistry study of more than 150 PC biopsies, Smyth *et al*. reported only an occasional positivity to CA IX immunostaining, even in the presence of hypoxic areas within the tumor [17]. The authors concluded that CA IX should not be considered a suitable marker of hypoxia in PC.

We have recently reported that hypoxia induces up-regulation of Notch 3 receptor and that Notch 3 expression correlates with the hypoxia-dependent markers, HIF-1α and CA IX, in human PC biopsies [18, 19]. We now analyze the hypoxia-induced response of CA IX in PC cell lines and extend the study of its expression to a larger panel of normal and diseased human prostate tissues, by immunohistochemistry.

## Methods

### Patients

For immunohistochemical analyses, 140 core needle biopsy specimens from 110 prostate cancer patients and 30 cancer-free patients were collected between January 2012 and September 2013, at the Urological Division of Siena University Hospital (Siena, Italy). Mean age at the time of biopsy was 70 years (range: 58 to 86 years); only patients without previous hormonal or radiation therapy were included in the study. Since the corresponding radical prostatectomy was available for each patient, the following clinic-pathological parameters were recorded: Gleason score and 2015 International Society of Urological Pathology (ISUP) grading, surgical margins infiltration, extra-prostatic extension, seminal vesicles invasion, lymph node metastasis, TNM staging (based on the AJCC Cancer Staging Manual, seventh edition, 2010, Springer New York, inc) and recurrence.

### Antibodies and chemicals

Anti-GAPDH antibody was obtained from Cell Signaling Technology (EuroClone, Milan, Italy); anti-HIF-1α antibody was obtained from Novus Biologicals (DBA, Italy) and anti-CA-IX (M75) antibody from Bioscience (Slovakia S.R.O.) [20]. Horseradish peroxidase (HRP)-conjugated secondary antibodies were purchased from Pierce Biotechnology Inc. (EuroClone, Milan, Italy). All other reagents were obtained from Sigma unless otherwise stated.

### Cell cultures

The androgen-dependent human prostate cancer cell line, LNCaP, and the androgen-independent cell line, PC-3, were obtained from ATCC (LGC Standards S.r.l., Italy), maintained in liquid nitrogen and used within few weeks after thawing and plating. Cells were grown in RPMI-1640 (EuroClone) (LNCaP) and DMEM (PC-3), supplemented with 10 % (vol/vol) fetal bovine serum (FBS) (EuroClone), L-glutamine (EuroClone) and 1 % (vol/vol) antibiotic/antimycotic solution (Gibco, Invitrogen S.r.l.). Hypoxia was achieved by maintaining the cells at 2 % oxygen, for 24 h, in a CO_2_ incubator (Forma Series II, Thermo Scientific) with oxygen sensor control, and CO_2_ and N_2_ gas regulators [18].

### Western blotting

For detection of CA IX, cells were lysed in cold buffer (10 mM TrisHCl pH 7.4, 25 mM MgCl2, 1 % Triton X-100, 1 mM dithiothreitol, 0.1 mM phenylmethylsulfonyl fluoride, 10 μg/ml leupeptin, 2 μg/ml aprotinin, 1 mM Na3VO4) and resolved by 10 % SDS-PAGE. CA IX immunoreactive bands were visualized by chemioluminescence (ECL) (GE Healthcare Italia, Euroclone, Milan, Italy). GAPDH was used for normalization of protein loading.

### Immunocytochemistry

Cells were grown under normoxia or hypoxia as previously described [18]. Immunocytochemistry was performed using the HRP multimer system (Ultra Vision Quanto Detector System, Thermo Scientific, Bio-Optica, Milan, Italy) and anti-CA-IX and anti-HIF-1α antibodies. 3,30-diaminobenzidine (DAB; Quanto, Thermo Scientific) was used as chromogen for the development of peroxidase activity (Hydrogen Peroxide Block Kit, Thermo Scientific).

### Histology and Immunohistochemistry

Core needle biopsy specimens were fixed in 10 % buffered formalin and embedded in paraffin; 4 μm-thick sections were cut from each block and stained with hematoxylin and eosin. Tumor pattern and score was established according to the modified Gleason grading system [21, 22] in each core needle biopsy, by two expert pathologists (MTdV and MRA) that reached a consensus in all cases. Tumors were classified as Gleason score 6, 7 (3 + 4 and 4 + 3), 8, 9 and 10, and grouped according to the recently proposed ISUP grading system for PC in grade 1 to 5 [23]. Staining was performed on 4+ 0.5 μm-thick sections of each block using the Ultravision Detection System Anti-Polyvalent HRP (Ultra V Block) (LabVision, Fremont, CA, USA, Bio-Optica). Slides were incubated with anti-CA-IX (dilution: 1:50) and anti-HIF-1α (dilution: 1:50) antibodies, using DAB as chromogen. Sections were weakly counterstained with Harris’ hematoxylin and examined under a light microscope. Non-immune serum immunoglobulins were used as negative controls. Clear cell renal cell carcinoma was used as positive control for both antibodies.

### Staining assessment

All samples were independently evaluated and scored by two investigators (BJR and AG). CA IX and HIF-1α protein expression was classified combining the percentage and the intensity of positively stained cells [24]. The percentage was scored as follows: 1 (<5 % positive cells), 2 (5–50 % positive cell), and 3 (>50 % positive cells). The intensity of staining was scored as follows: 1 (weak or not detectable staining), 2 (moderate staining) and 3 (strong staining). Three different fields (at least 100 cells/field) were evaluated at x200 magnification. The sum of the percentage score and the intensity staining score was used to define CA IX and HIF-1α protein expression as low (0–2) or high (3–4). The agreement between the two pathologists was about 90 %. Cases with discrepancies were reviewed and discussed to reach 100 % concordance. In Gleason score 7 and 9 samples, staining assessment was performed separately in the two patterns (i.e. 3 and 4 in Gleason score 7, and 4 and 5 in Gleason score 9).

### Statistical analysis

Statistical analysis was performed using a statistical software package (SigmaPlot 12.0, Systat Software). The correlation between CA IX and HIF-1α expression, and Gleason and 2015 ISUP grading was evaluated by the Spearman Rank Order Correlation Test. When computable, the Fisher exact test was applied to contingency tables to evaluate the association between the frequency distribution of CA IX and each qualitative variable. We alternatively used the classic Chi-square test. A *p* < 0.05 was considered statistically significant.

## Results and discussion

### Expression of CA IX is up-regulated by hypoxia and is mainly nuclear in PC cells

**Fig. 1:**
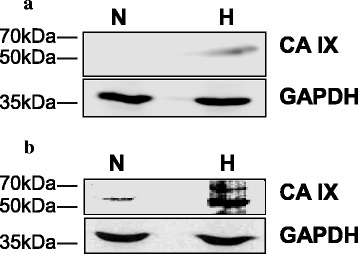
Western blot analysis of CA IX in LNCaP and PC-3 cells, under normoxia and hypoxia. Low protein expression is detected in LNCaP (**a**) and PC-3 (**b**) cells under normoxia (N). Under hypoxia (H) the staining becomes evident and is stronger in PC-3 than LNCaP cells. GAPDH is used as control for protein loading

**Fig. 2:**
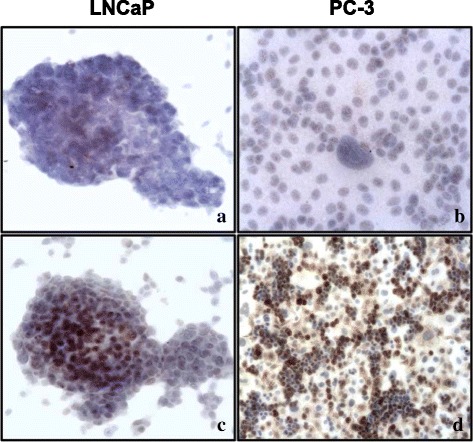
Immunocytochemical analysis of CA IX in LNCaP and PC-3 cells, under normoxia and hypoxia. Under normoxia, a negligible CA IX cytoplasmic staining is detected in LNCaP (**a**) and PC-3 (**b**) cells, with few positive nuclei. In response to hypoxia, a strong positivity for CA IX appears in the cytoplasm and in the nucleus of LNCaP (**c**) and PC-3 cells (**d**) (**a**-**d**, original magnification (O.M.): 400x)

**Fig. 3:**
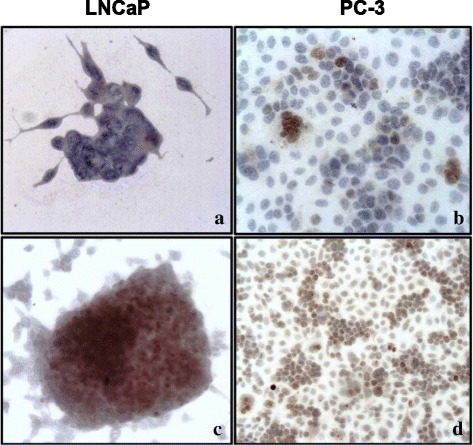
Immunocytochemical analysis of HIF-1α in LNCaP and PC-3 cells, under normoxia and hypoxia. No staining or mild cytoplasmic positivity for HIF-1α is detected in LNCaP (**a**) and PC-3 (**b**) cells, under normoxia. Under hypoxia, cytoplasmic positivity becomes stronger, both in LNCaP (**c**) and PC-3 cells (**d**). Nuclear translocation of the protein is also evident (**c**-**d**) (**a**-**d**, O.M.: 400x)

Fiaschi *et al*. [10] have shown the expression of CA IX in several PC cell lines. We tested whether its expression was up-regulated by hypoxia in LNCaP and PC-3 cells. Under normoxia, the enzyme was expressed very poorly in LNCaP cells but was easily detected in PC-3 cells. Under hypoxia, CA IX expression was up-regulated in both cell lines (Fig. 1). This is not surprising considering that CA IX is a well-known hypoxia-induced gene [25, 26]. Remarkably, when the expression of CA IX was analyzed by immunocytochemistry, a low cytoplasmic staining was detected in scattered cells, with some positive nuclei (Fig. 2a-b). In response to hypoxia, the staining became stronger and the protein was expressed mainly in the nucleus (Fig. 2c-d). The intensity of the staining was higher in PC-3 cells, both under normoxia and hypoxia (Fig. 2b-d). Positivity for HIF-1α confirmed the effectiveness of the hypoxia protocol (Fig. 3).

### Expression of CA IX and HIF-1α in PC biopsies correlates with Gleason pattern and score

**Fig. 4:**
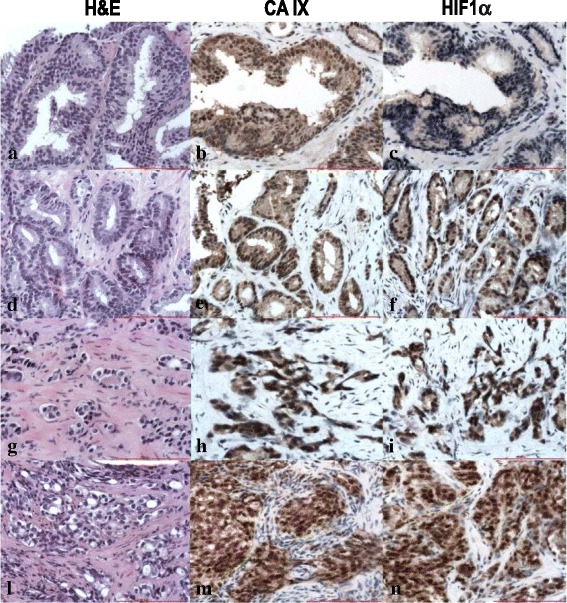
Immunohistochemical analysis of CA IX and HIF-1α in HGPIN and Gleason pattern 3 to 5. Low cytoplasmic staining for CA IX and HIF-1α in HGPIN (**a**-**c**); low-to-moderate cytoplasmic staining, with few positive nuclei, for CA IX and HIF-1α in Gleason pattern 3 (**d**-**f**); strong cytoplasmic and nuclear expression of CA IX and HIF-1α in Gleason pattern 4 (**g**-**i**) and 5 (**l**-**n**) (**a**,**d**,**g**,**l**: hematoxylin and eosin (H&E); **b**,**e**,**h**,**m**: CA IX staining; **c**,**f**,**i**,**n**: HIF 1α staining. O.M. 200x)

**Fig. 5:**
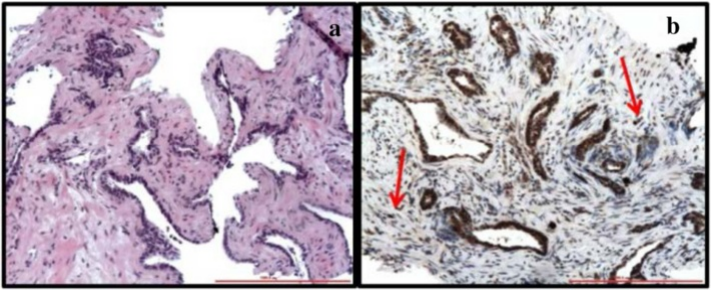
Immunohistochemical analysis of CA IX in cancer-free specimens. Low CA IX staining in atrophic glands with higher expression in intermingled stromal (*arrows*) and endothelial cells (**a**, H&E; **b**, CA IX stain; **a**-**b**. O.M.: 200x)

We analyzed 30 non neoplastic specimens (18 with atrophy and 12 with atrophy *plus* chronic inflammation) and 110 cancer biopsies, from high grade prostate intraepithelial neoplasm (HGPIN) (*N* = 20), to Gleason score 6 (*N* = 20), Gleason score 7 (*N* = 20), Gleason score 8 (*N* = 30), Gleason score 9 (*N* = 10), and Gleason score 10 (*N* = 10). In HGPIN, a low cytoplasmic staining for CA IX and HIF-1α was observed, with scattered positive nuclei (Fig. 4a-c). CA IX and HIF-1α protein expression was lower in low Gleason pattern (i.e. 3) (Fig. 4d-f) compared to high Gleason pattern (i.e. 4 and 5) (Fig. 4g-i and l-n, respectively). In Gleason pattern 3, CA IX positivity was mainly cytoplasmic, whereas in Gleason pattern 4 and 5, the enzyme was detected in the cytoplasm and in the nucleus of all neoplastic cells. In addition, CA IX and HIF-1α immunostaining was significantly lower in Gleason score 6 than in Gleason score 8 to 10. In Gleason score 7, an intra-tumoral heterogeneity was observed, with a higher expression level of both proteins in Gleason pattern 4 than in Gleason pattern 3. These findings were better exemplified by using the novel 2015 ISUP grading system for PC that highlights how Gleason score 3 + 4 = 7 really is a different form of histology compared to Geason score 4 + 3 = 7. We found that 3 to 5 PC grade groups highly expressed CA IX and HIF-1α in comparison to grade groups 1–2. The correlation between the level of expression of CA IX and HIF-1α, and Gleason (pattern and score) and ISUP grading was statistically significant (*p* < 0.001) and is summarized in Tables 1, 2, 3 and 4. No staining was detected in non-neoplastic specimens for both antibodies, except for 8 samples with atrophy showing a low level of CA IX expression (Fig. 5). Stromal and endothelial cells intermingled with non-neoplastic glands demonstrated significant CA IX positivity (see arrows). The intensity of expression of CA IX directly correlated with TNM stage (*p* < 0.001), surgical margins infiltration (*p* < 0.001), extra-prostatic extension (*p* < 0.001), seminal vesicle invasion (*p* < 0.001) and lymph node metastasis (*p* < 0.001). Interestingly the patients with higher CA IX expression recurred more frequently than those with lower level. However, a longer follow-up is mandatory to assess the real impact of the protein expression on patients’ outcome.

**Table 1:** CA IX expression and Gleason score

Gleason score	N	High	Low
6	20	5	15
7	20	12	8
8	30	27	3
9	10	9	1
10	10	10	0

N: number of cases

*p* < 0.001

**Table 2:** HIF-1α expression and Gleason score

Gleason score	N	High	Low
6	20	3	17
7	20	11	9
8	30	25	5
9	10	9	1
10	10	9	1

N: number of cases

*p* < 0.001

**Table 3:** CA IX expression and 2015 ISUP grading system

ISUP grading	N	High	Low
1	20	5	15
2	10	3	7
3	10	8	2
4	30	27	3
5	20	18	2

N: number of cases

*p* < 0.001

**Table 4:** HIF-1α expression and 2015 ISUP grading system

ISUP grading	N	High	Low
1	20	3	17
2	10	2	8
3	10	9	1
4	30	25	5
5	20	18	2

N: number of cases

*p* < 0.001

CA IX is an attractive target for innovative anti-cancer therapies due to its role in promoting survival of cancer cells in inhospitable microenvironments. Since the discovery of isoform IX [1], the primary scientific interest has revolved around its membrane-bound enzymatic activity which regulates extra and intracellular pH and sustains cancer cell migration, metastatization and tumor progression [10]. Only in recent years, it became apparent that CA IX likely plays additional roles inside the cell. Those roles are recapped by the presence of the protein in different sub-cellular compartments. A strong CA IX cytoplasmic staining was detected in squamous cell head and neck cancers and was related to poor response to chemotherapy [27, 28]. In non-small-cell lung cancer, perinuclear CA IX was an independent poor prognostic marker [29]. In neuroblastoma, nuclear CA IX expression was significantly higher in patients with adverse clinical and pathological features [30]. In PC tissues, the unconventional location of the protein is quite striking. In a limited series of human biopsies, we previously demonstrated that CA IX is mainly cytoplasmic in low grade carcinomas, whereas it is strongly expressed in the cytoplasm and in the nucleus of high grade tumor cells [19]. We have now extended this observation to a larger series of human PC biopsies, confirming our previous findings. Unlike Smyth *et al*. that reported only an occasional CA IX positivity in hypoxic areas [17], we found a statistically significant association between CA IX and HIF-1α expression in PC that identifies the protein as a reliable marker of tumor hypoxia.

Hypoxia orchestrates a composite intracellular response in tumor cells. HIF-1α induces the expression of several other factors, including VEGF, lysyl oxidase (LOX) and CA IX, all potentially relevant in the context of PC biology [31]. However, how the interaction between these factors modulates the progression of the tumor or what is the combination of markers that better predicts an unfavorable outcome is still undetermined [32]. Since CA IX represents a marker of hypoxia and since hypoxia induces resistance to radiotherapy, it is reasonable that high level of the protein may impact the response to therapy in muscle-invasive PC.

We found a positive correlation between CA IX expression and the most relevant prognostic factors of PC (Gleason score, ISUP 2015 grading system, TNM stage). Indeed, the nuclear localization of the enzyme may epitomize a biological switch that stirs the tumor towards a worst outcome. The limited expression of CA IX in LNCaP cells, compared to PC-3 cells, is in line with the association of the enzyme with malignancy. Fiaschi *et al*. demonstrated that CA IX is expressed by prostate cancer-associated fibroblasts, upon their activation by cancer-delivered soluble factors, and that the enzyme mediates the epithelial-mesenchymal transition of cancer cells, with subsequent increased motility, survival and tumor stemness [10]. These data further support a central role of CA IX in PC progression. Further studies will be necessary to elucidate whether CA IX represents an independent poor prognostic marker in PC patients.

Recently, Buanne *et al*. [33] designed the “interactomic” map of CA IX, demonstrating that the majority of partners were proteins that belong to the nuclear transport machinery. These data support the notion that CA IX is able to transit through the nuclear compartment. They also found that hypoxia increases CA IX nuclear accumulation [33]. The study also indicated that the peptide detected in the nuclear/perinuclear region encompasses the extracellular, catalytic domain, likely representing an endocytosed fraction of the full-length transmembrane protein [33, 34]. Our data also indicate that nuclear-bound CA IX encompasses the N-terminal extracellular region, being detected by a monoclonal antibody directed against the proteoglycan-like domain. Whether nuclear-bound CA IX has a role in cellular signalling and/or regulation of transcription is still unclear. However, it is interesting that a transcription factor with CA activity has been identified in human tissues, suggesting that CA IX may act as a transcription factor [35]. In any case, the possibility that cytoplasmic/nuclear-localized CA IX may have distinct biological functions than the membrane-bound protein should be acknowledged.

## Conclusion

CA IX is readily detected in PC tissues and correlates with the expression of HIF-1α, defining the protein a suitable marker of hypoxia in this tumor. Moreover, the expression of CA IX correlates with Gleason grade and score, indicating that the enzyme may contribute to tumor progression. Cytoplasmic/nuclear-bound CA IX may display previously unrecognized, alternative biological functions that should be defined further before the enzyme may be considered as a target for novel anti-cancer therapies.

## Abbreviations

CA: carbonic anhydrase
CARP: CA-related proteins
ECL: chemioluminescence
DAB: diaminobenzidine
ECD: ectodomain
FBS: fetal bovine serum
HGPIN: high grade PIN
HRP: horseradish peroxidase
HIF: hypoxia-inducible factor
PC: prostate cancer
pVHL: von Hippel-Lindau
